# Identification of Candidate Parkinson Disease Genes by Integrating Genome-Wide Association Study, Expression, and Epigenetic Data Sets

**DOI:** 10.1001/jamaneurol.2020.5257

**Published:** 2021-02-01

**Authors:** Demis A. Kia, David Zhang, Sebastian Guelfi, Claudia Manzoni, Leon Hubbard, Regina H. Reynolds, Juan Botía, Mina Ryten, Raffaele Ferrari, Patrick A. Lewis, Nigel Williams, Daniah Trabzuni, John Hardy, Nicholas W. Wood

**Affiliations:** 1 Department of Molecular Neuroscience, University College London Institute of Neurology, Queen Square, London, United Kingdom; 2 Department of Pharmacology, School of Pharmacy, University College London, London, United Kingdom; 3 Division of Psychological Medicine and Clinical Neurosciences, School of Medicine, University of Cardiff, Cardiff, United Kingdom; 4 Departamento de Ingeniería de la Información y las Comunicaciones, Universidad de Murcia, Murcia, Spain; 5 Department of Comparative Biomedical Sciences, The Royal Veterinary College, London, United Kingdom; 6 School of Pharmacy, University of Reading, Reading, United Kingdom; 7 Department of Genetics, King Faisal Specialist Hospital and Research Centre, Riyadh, Saudi Arabia; 8 Department of Neurodegenerative Disease and Reta Lila Weston Laboratories, University College London Dementia Research Institute, London, United Kingdom

## Abstract

**Importance:**

Substantial genome-wide association study (GWAS) work in Parkinson disease (PD) has led to the discovery of an increasing number of loci shown reliably to be associated with increased risk of disease. Improved understanding of the underlying genes and mechanisms at these loci will be key to understanding the pathogenesis of PD.

**Objective:**

To investigate what genes and genomic processes underlie the risk of sporadic PD.

**Design and Setting:**

This genetic association study used the bioinformatic tools Coloc and transcriptome-wide association study (TWAS) to integrate PD case-control GWAS data published in 2017 with expression data (from Braineac, the Genotype-Tissue Expression [GTEx], and CommonMind) and methylation data (derived from UK Parkinson brain samples) to uncover putative gene expression and splicing mechanisms associated with PD GWAS signals. Candidate genes were further characterized using cell-type specificity, weighted gene coexpression networks, and weighted protein-protein interaction networks.

**Main Outcomes and Measures:**

It was hypothesized a priori that some genes underlying PD loci would alter PD risk through changes to expression, splicing, or methylation. Candidate genes are presented whose change in expression, splicing, or methylation are associated with risk of PD as well as the functional pathways and cell types in which these genes have an important role.

**Results:**

Gene-level analysis of expression revealed 5 genes (*WDR6* [OMIM 606031], *CD38* [OMIM 107270], *GPNMB* [OMIM 604368], *RAB29* [OMIM 603949], and *TMEM163* [OMIM 618978]) that replicated using both Coloc and TWAS analyses in both the GTEx and Braineac expression data sets. A further 6 genes (*ZRANB3* [OMIM 615655], *PCGF3* [OMIM 617543], *NEK1* [OMIM 604588], *NUPL2* [NCBI 11097], *GALC* [OMIM 606890], and *CTSB* [OMIM 116810]) showed evidence of disease-associated splicing effects. Cell-type specificity analysis revealed that gene expression was overall more prevalent in glial cell types compared with neurons. The weighted gene coexpression performed on the GTEx data set showed that *NUPL2* is a key gene in 3 modules implicated in catabolic processes associated with protein ubiquitination and in the ubiquitin-dependent protein catabolic process in the nucleus accumbens, caudate, and putamen. *TMEM163* and *ZRANB3* were both important in modules in the frontal cortex and caudate, respectively, indicating regulation of signaling and cell communication. Protein interactor analysis and simulations using random networks demonstrated that the candidate genes interact significantly more with known mendelian PD and parkinsonism proteins than would be expected by chance.

**Conclusions and Relevance:**

Together, these results suggest that several candidate genes and pathways are associated with the findings observed in PD GWAS studies.

## Key Points

**Question:**

What genes and genomic processes underlie risk of sporadic Parkinson disease?

**Findings:**

This genetic association study integrated Parkinson disease genome-wide association study data and brain-derived gene regulation data using various complementary bioinformatic tools and identified 11 candidate genes with evidence of disease-associated regulatory changes. Coexpression and protein level analyses of these genes demonstrated a significant functional association with known mendelian Parkinson disease genes.

**Meaning:**

This study suggests that gene regulation data may be used to identify candidate genes and pathways involved in sporadic Parkinson disease.

## Teaser

This genetic association study investigates what genes and genomic processes underlie the risk of sporadic Parkinson disease.

## Introduction

Parkinson disease (PD) is the second most common neurodegenerative condition worldwide, characterized by bradykinesia, rigidity, and tremor.^1^ Genome-wide association studies (GWASs) have discovered more than 40 loci significantly associated with PD risk.^2^ However, these studies are limited to narrowing PD risk down to a genomic region encompassing several candidate genes. The causal genes underlying each locus, as well as the mechanism by which they confer PD risk, often remain unclear. These issues can be resolved by complementing GWAS data with quantitative trait loci (QTL) data sets, which describe associations of an individual’s genotype with gene expression (eQTLs), splicing or methylation. For example, such efforts applied to the 7p15.3 locus have shown that PD risk variants regulate *GPNMB* (OMIM 604368) expression.^3^ Improvements to the understanding of the genes and mechanisms via which GWAS risk variants act may be instrumental to our understanding of the pathogenesis of PD.

Several studies demonstrate that GWAS risk variants may have an association with gene expression or splicing.^4,5^ In recent years, there has been an increase in the number of publicly available brain-derived QTL data sets, including those released by the UK Brain Expression Consortium and the Genotype-Tissue Expression (GTEx) Consortium used in this study.^6,7,8^ The progressively increasing sample size, sequencing depth, and tissue resolution of these data sets improves our power to detect QTLs and, as a result, to interpret GWAS results in the context of regulatory effects.^9,10^ This improved resource availability has been accompanied by advances in statistical tools that permit the systematic, genome-wide integration of QTL and GWAS data. Because different methods have different limitations and assumptions, we adopted a stringent approach of applying the tools Coloc and transcriptome-wide association study (TWAS), then retaining only significant results present from both methods to reduce the likelihood of false-positive results. Coloc is a method that uses a bayesian framework to calculate a probability that 2 traits share a causal variant, whereas TWAS uses prediction models trained on reference QTL data to assess the association between gene expression and disease.^11,12,13^ The increased size and comprehensiveness of publicly available brain QTL data sets, coupled with advancements in bioinformatic tools, allow for a more thorough investigation of which of the putative genes suggested by GWAS underlie PD risk.

We present a systematic interrogation of all known PD risk loci using a recent PD GWAS, the aforementioned data sets, and methods to uncover the genes and mechanisms through which GWAS risk variants are associated with PD risk. The candidate genes and resulting pathways presented in this study are the result of stringent replication across multiple data sets and bioinformatic methods. We anticipate that this work can serve as a reference example to be applied to different neurodegenerative diseases.

## Methods

### PD GWAS Data

Summary statistics from the combined discovery and replication phases of a GWAS meta-analysis of PD were used,^2^ including 8 055 803 genotyped and imputed variants in up to 26 035 patients with PD and 403 190 controls of European ancestry. For the purposes of this study, all alleles were aligned on the forward strand, and all effect sizes and allele frequencies were converted with respect to the nonreference allele in build GRCh37. All genes overlapping the region 1 Mb upstream or downstream of a single-nucleotide variation (SNV) with an association with PD of *P* ≤ 5 × 10^−8^ were selected for the initial analysis. The analysis was then extended to include all genes in the genome, to identify candidate genes in loci that have not reached genome-wide significance in the PD GWAS but where the collective evidence with expression data suggests a colocalized signal.

### Braineac eQTL Data

The UK Brain Expression Consortium Braineac data set contains data from 10 brain regions obtained from 134 control individuals: frontal cortex, temporal cortex, occipital cortex, hippocampus, thalamus, putamen, substantia nigra, medulla, cerebellum, and white matter, together with the mean expression across all 10 regions.^6,8,14^ Gene expression was quantified using Affymetrix Exon 1.0 ST arrays (Thermo Fisher Scientific), and the genotyping was performed using Illumina Infinium Human Omni1-Quad BeadChip microarrays (Illumina Inc), then imputed to the European panel of the phase 1 1000 Genomes Project.^15,16^ The genotyped and imputed data were restricted to approximately 5.88 million SNVs with minor allele frequency of 0.05 or more and imputation *r*^2^ > 0.5. For each gene of interest, all SNV associations within 1 Mb upstream and downstream of the gene were collected.^17^

### GTEx eQTL Data

The GTEx version 7 data set includes eQTL data from 13 brain tissues with sample sizes ranging from 154 to 80: cerebellum, caudate, cortex, nucleus accumbens, cerebellar hemisphere, frontal cortex, putamen, hippocampus, anterior cingulate cortex, hypothalamus, amygdala, spinal cord (cervical C1), and substantia nigra.^7^ Gene expression in these samples has been obtained using paired-end RNA-seq (Illumina TruSeq; Illumina Inc) and genotype data from whole-genome sequencing. Full-summary eQTL data for the tissues of interest were downloaded from the GTEx web portal.^18^

### Brain Methylation Data

Genome-wide methylation profiles were obtained from both the substantia nigra and the frontal cortex of 134 individuals with PD from the Parkinson Disease UK Brain Bank, using the Illumina Infinium HumanMethylation450 BeadChip (Illumina Inc). *Cis* PD methylation quantitative trait loci were defined as correlations between the target PD SNV genotype and DNA methylation levels of CpG sites within a 500-kb window of the SNV base position. Linear models were fitted to test whether DNA methylation beta values for each CpG site were predicted by SNV genotypes. We included covariates for age at death, sex, population stratification, batch, and postmortem interval. We retained the strongest SNV-CpG pair at a 5% false discovery rate (FDR) to be used in downstream analyses. The CpG sites were mapped to genes if they were within 10 kb of the gene transcription start or end base position according to HG19 (human genome version 19) coordinates. In total, 37 460 CpG sites were included in the final analysis.

### Coloc Analysis

To assess the probability of the same SNV being responsible for both changing PD risk and modulating the expression levels of a gene, we used the Coloc method.^11^ Both the Braineac and GTEx eQTL data sets were harmonized with the PD GWAS data set to ensure that the regression coefficients were reported with respect to the nonreference alleles in build GRCh37 and that the variants overlapping with the PD GWAS data set were kept for analysis. Coloc uses estimated approximate Bayes factors from summary association data to compute posterior probabilities for the following 5 hypotheses: no shared causal variant in the region, there is a causal PD variant but no eQTL variant, there is a causal eQTL variant but no PD variant, both studies have a different causal variant within the analyzed region, and there is a shared causal variant within the analyzed region.

We used the default Coloc priors of p1 = 10^−4^, p2 = 10^−4^, and p12 = 10^−5^, where p1 is the probability that a given SNV is associated with PD, p2 is the probability that a given SNV is a significant eQTL, and p12 is the probability that a given SNV is both a PD result and an eQTL.

For both the Braineac and GTEx data sets, we derived posterior probabilities (PPH0-4) for each gene and considered PPH4 of 0.75 or more as strong evidence for colocalization. For Braineac, we also looked at genes for which there is strong evidence of colocalization at the exon level for a given exon (exon PPH4 ≥ 0.75), but evidence against colocalization for the whole gene (gene PPH3>gene PPH4), to identify potential splicing events causing PD.

### Transcriptome-Wide Association Study

To assess the degree to which changes in gene expression or splicing might be associated with PD case or control status, we performed a TWAS or methylation-wide association study (MWAS) using the method by Gusev et al.^12^ Expression reference weights were obtained from the CommonMind Consortium dorsolateral prefrontal cortex RNA-seq and RNA-seq splicing data sets, which are based on 467 samples (209 individuals with schizophrenia, 206 controls, and 52 individuals with affective disorder), and methylation data from our PD brain methylation data set.^19^ For all genes (or isoforms for splicing analysis), TWAS and MWAS *P* values were obtained, and all genes and isoforms passing multiple testing correction at the FDR 0.05 level, genome-wide, were considered significant. Where multiple genes were implicated within a region, we performed further conditional analyses using Fusion^12^ to identify whether there were single or joint TWAS and MWAS signals at each locus. Conditional analyses were performed independently across gene expression and methylation data sets.

### Weighted Gene Coexpression Network Analysis

Weighted gene coexpression network analysis (WGCNA) with k-mean values was applied by Botía et al^20^ to transcriptomic data from GTEx and Braineac to generate coexpression modules.^21,22^ We assessed whether the candidate genes in this study were important in certain modules using the tool CoExpNets.^23^ The GTEx- and Braineac-derived networks were used to assign each candidate gene to a cell type, while the GTEx networks alone were used to assess the functional pathways associated with each gene. In brief, each module is associated with a cell type based on the enrichment of cell-type–specific genes within the module. The enrichment is assessed by using the Fisher exact test to evaluate whether we can find an overlap between the module genes and the brain cell–type markers that is more significant than random chance. Each gene of interest is then assigned to a primary cell type based on its module membership, which is the correlation of the expression of our gene of interest with the first principal component of each module. This correlation is always between 0 and 1; we use module membership as a measure of how reliable the assignment is of each gene to its module.

### Cell-Type Specificity Analysis

We investigated the cell-type–specific expression of the Coloc prioritized genes, using the immunopanning data from humans and mice, and coexpression analysis of the GTEx and Braineac data.^15,16,24^ From the immunopanning data, cell-type–specific enrichment values were obtained or calculated for each gene and for each cell type analyzed. Enrichment was calculated as expression prevalence by dividing the mean expression of the gene in one cell type by the mean expression across all other cell types. Each gene of interest was then assigned to a primary cell type of interest based on the highest cell-type–specific enrichment value observed.

### Literature-Derived Protein Interactor Networks

We extracted and quality-controlled currently known protein interactors (PPIs) for the proteins (seeds) encoded by the genes prioritized in this article (Coloc protein network), using a previously described custom-weighted protein-protein interaction (WPPINA) pipeline (eAppendix in Supplement 1), and performed functional enrichment analysis on the relevant genes prioritized through the WPPINA.^25^ For comparison, a similar network (mendelian protein network) was prepared for mendelian PD and parkinsonism genes (*SNCA* [OMIM 163890], *LRRK2* [OMIM 609007], *GBA* [OMIM 606463], *SMPD1* [OMIM 607608], *VPS35* [OMIM 601501], *DNAJC13* [OMIM 614334], *PINK1* [OMIM 608309], *PRKN* [OMIM 602544], *DJ1* [OMIM 602533], *FBX07* [OMIM 605648], *SYNJ1* [OMIM 604297], *DNAJC6* [OMIM 608375], *WDR45* [OMIM 300526], *PLA2G6* [OMIM 603604], *ATP13A2* [OMIM 610513], *RAB39B* [OMIM 300774], *SPG11* [OMIM 610844], *PANK2* [OMIM 606157], *C19orf12* [OMIM 614297], and *PRKRA* [OMIM 603424]; eTable 1 in Supplement 2). The number of interactions between the Coloc protein network and the mendelian protein network were quantified. To assess the probability that the number of connections between the 2 networks was more than would be expected by chance, we performed random simulations with 1000 control networks characterized by the same number of seeds as the Coloc protein network. Control networks were built by using random combinations of seed genes sampled out of the pool of 118 genes characterized by evidence against colocalization in the Coloc analysis (PPH3 > 0.75).

### Statistical Analysis

Data wrangling and analysis were performed using R, version 3.4.3 (R Foundation for Statistical Computing). Coloc analyses were performed using the Coloc package in R. Transcriptome-wide association study analyses were conducted using the TWAS software,^26^ and WGCNA results were obtained using the R package.^23^ Cell-type specificity was calculated using in-house R scripts detailed in the Methods. The WPPINA analysis was performed using R, with the results visualized using Cytoscape 3.5.0 software (Cytoscape Consortium) and functional enrichment using g:Profiler.^27^ The code for analyses performed in this study is available online.^28^

## Results

### Genes Associated With PD Risk Through Changes in Expression

First, to evaluate the scope of the analysis, we assessed the number of genes expressed to a detectable level in both the Braineac and GTEx data sets. We found that of 515 genes within 1 Mb of a significant PD risk variant, 470 were expressed to a detectable level and passed quality control in both data sets. Second, we applied Coloc and TWAS to test whether the regulation of the expression of these overlapping genes was associated with PD risk. When applying the Coloc method to these 470 genes, 9 in Braineac and 27 in GTEx showed strong evidence for colocalization (PPH4 ≥ 0.75) in at least 1 brain region. In the TWAS analysis, 61 genes were found to be significantly associated with PD risk at an FDR level of 0.05. Five genes *(WDR6* [OMIM 606031], *CD38* [OMIM 107270], *GPNMB* [OMIM 604368], *RAB29* [OMIM 603949], and *TMEM163* [OMIM 618978]; Table 1) replicated across the Coloc and TWAS results (eFigure 1A in Supplement 1). Example regional association plots for the genes with the highest PPH4 in Braineac (*RAB29*) and GTEx (*CD38* in putamen) are found in eFigure 2A and B in Supplement 1. Full details for all genes that showed strong evidence for colocalization in either Braineac or GTEx and were significant in the TWAS analysis are found in eTable 2 in Supplement 3 and eTable 3 in Supplement 4.

**Table 1.** Gene-Based Results

Gene	Locus	Direction	Top PD SNV position
*WDR6*	*NCKIPSD* or *CDC71*	Negative	chr3:48748989
*CD38*	*FAM200B* or *CD38*	Negative	chr4:15737101
*GPNMB*	*KLHL7*, *NUPL2*, or *GPNMB*	Positive	chr7:23293746
*RAB29*	*NUCKS1* or *SLC41A1*	Positive	chr1:205723572
*TMEM163*	*TMEM163* or *CCNT2*	Positive	chr2:135539967

Abbreviations: PD, Parkinson disease; SNV, single-nucleotide variation.

### Genes Associated With PD Risk Through Changes in Splicing

To assess splicing changes, we used the Braineac data set, which had exon-level eQTL data. A total of 25 genes in Braineac had strong evidence for colocalization for at least 1 exon in at least 1 brain region. For 15 genes there was evidence suggesting that the association is owing to an exon-level splicing event (exon PPH4 ≥ 0.75) rather than a gene-level expression effect (gene PPH3>PPH4). In the TWAS analysis, 129 genes had evidence for splicing in at least 1 isoform at FDR 0.05 level. Of these, 40 were within 1 Mb of a PD-significant SNV. Six genes with a putative splicing effect in the Coloc analysis showed a significant splicing effect in the TWAS analysis (*ZRANB3* [OMIM 615655], *PCGF3* [OMIM 617543], *NEK1* [OMIM 604588], *NUPL2* [NCBI 11097], *GALC* [OMIM 606890], and *CTSB* [OMIM 116810]) (eFigure 1B in Supplement 1). We then assessed the eQTL *P* values of the top SNV suggested by Coloc for the associated exon and the gene as a whole, showing that, for these associations, the gene-level *P* value is not significant, while the exon-level *P* value is significant. These genes are summarized in Table 2. Full details for all splicing events that showed strong evidence for colocalization in Braineac, and of all genes significant at FDR 0.05 level in the TWAS analysis are found in eTable 4 in Supplement 5 and eTable 5 in Supplement 6.

**Table 2.** Splicing Results

Gene	PPH3	PPH4	Top Coloc SNV	*P* value		PPH3	PPH4
				Exon	Gene		
*ZRANB3*	0.06	0.93	rs6741007	1.37 × 10^−6^	.27	0.20	0.07
*PCGF3*	0.05	0.87	rs34311866	3.32 × 10^−5^	.20	0.26	0.07
*NEK1*	0.11	0.78	rs6828248	7.13 × 10^−5^	.11	0.28	0.08
*NUPL2*	0.24	0.76	rs12539467	8.62 × 10^−9^	.06	0.26	0.17
*GALC*	0.12	0.81	rs2008686	7.7 × 10^−5^	.03	0.27	0.19
*CTSB*	0.10	0.78	rs1692821	3.14 × 10^−5^	.47	0.27	0.05
*CTSB*	0.09	0.77	rs1293298	3.66 × 10^−5^	.08	0.27	0.07

Abbreviations: PPH, posterior probabilities; SNV, single-nucleotide variation.

### Genes Associated With PD Risk Through Changes in Methylation

We investigated whether the genes associated with PD risk via expression or splicing changes could be acting through regulation of methylation. A total of 134 CpG sites passed FDR correction and conditional analysis in the substantia nigra (mapping to 107 unique genes), and 116 CpG sites survived FDR correction and conditional analysis in substantia nigra (mapping to 93 unique genes) (eTable 6 in Supplement 7). Of the MWAS significant genes, 3 (*GPNMB*, *TMEM163*, and *CTSB*) overlapped with the Coloc expression or splicing results (Table 3).

**Table 3.** MWAS Results Overlapping With Coloc Hits

Gene	CpG site	Direction
*GPNMB*	cg17274742	Negative
*GPNMB*	cg08455073	Negative
*TMEM163*	cg00897703	Positive
*CTSB*	cg07593977	Negative

Abbreviation: MWAS, methylation-wide association study.

### Cell-Type Specificity and WGCNA

**Figure 1.**
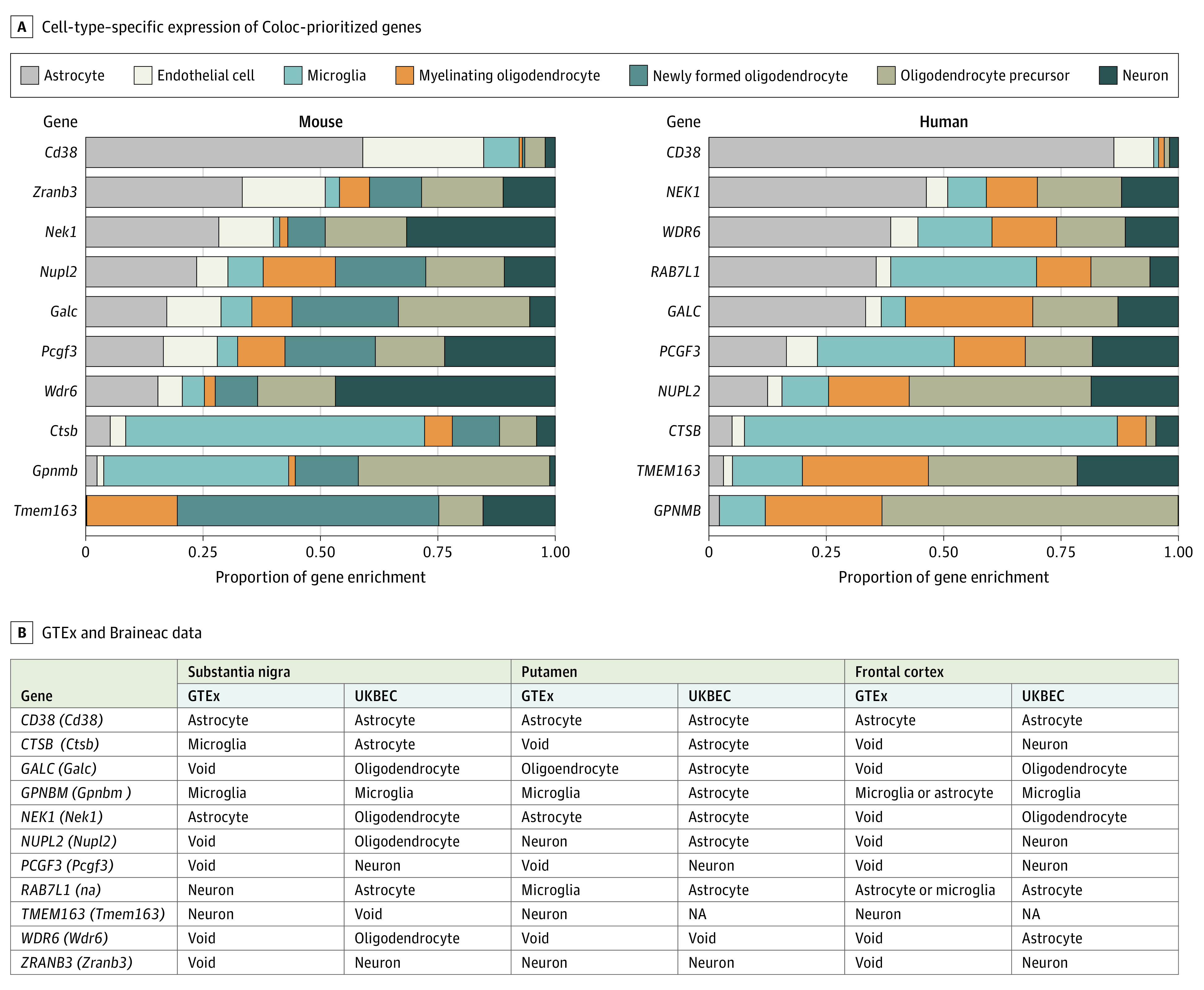
Cell-Type–Specific Expression of Coloc-Prioritized Genes in Human and Mouse, and Using Genotype-Tissue Expression (GTEx) Consortium and United Kingdom Brain Expression Consortium (UKBEC) Data These results illustrate the overrepresentation of glial cell types compared with neuronal cell types among the candidate genes. NA indicates not applicable.

The results for the cell-type specificity analysis are shown in Figure 1. Although no single cell type dominated, Coloc-prioritized gene expression was overall more prevalent in glial cell types compared with neurons. This finding was consistent across analyses performed with mouse immunopanning data generated from the cortex and with human immunopanning data generated using cortical tissue and using inferred cell-specific gene expression generated using coexpression networks across all brain regions, including the substantia nigra. The WGCNA results are summarized in Figure 1B; *NUPL2*, *TMEM163*, and *ZRANB3* were the most relevant genes (module membership >0.76) within 3 modules in different brain regions. *NUPL2* was a key gene within the dark turquoise module in the nucleus accumbens, the blue module in the caudate, and the sky blue module in the putamen. These modules’ most relevant functions indicated catabolic processes associated with protein ubiquitination (protein ubiquitination [gene ontology (GO): 0016567]; ubiquitin-dependent protein catabolic process [GO: 0006511]). *TMEM163* and *ZRANB3* were both important in the turquoise module in the frontal cortex and caudate, respectively. This module indicated chemical transmission at the synapse as a major associated function (regulation of signaling [GO: 0023051]; cell communication [GO: 0007154]).

### Literature-Derived PPI Network

**Figure 2.**
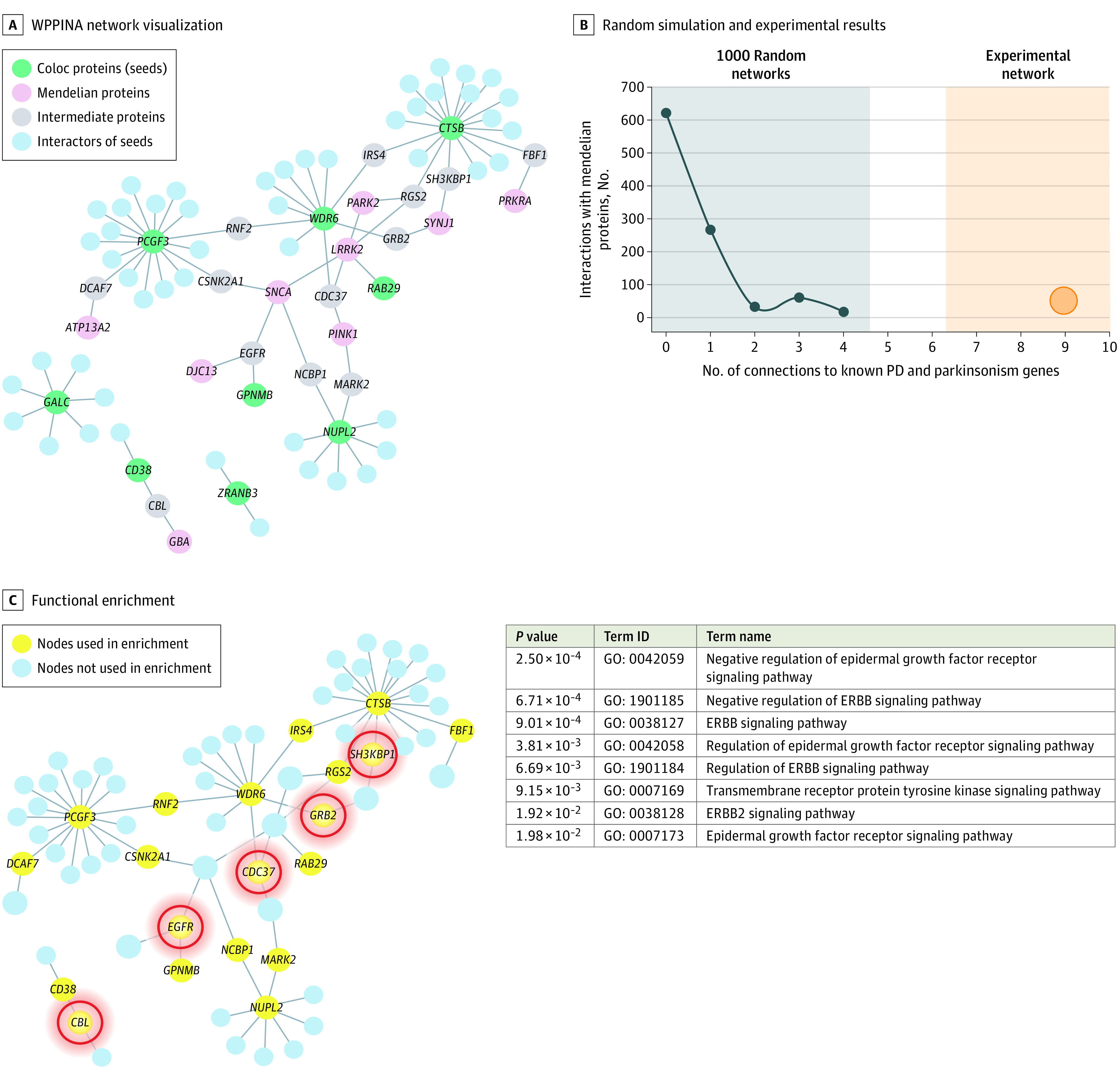
Literature-Derived Protein-Protein Interaction (PPI) Network A, Weighted protein-protein interaction network analysis (WPPINA) network visualization of the PPIs specific for the proteins (seeds) coded by the Coloc genes (green nodes). Minor protein interaction partners are shown in blue, while mendelian Parkinson and parkinsonism proteins interacting with parts of the seeds’ interactome are reported in pink. Major interaction partners (ie, they bridge interaction between at least a Coloc protein and a mendelian protein) are labeled in gray. B, The negative control protein network has been randomly sampled to generate 1000 random networks with similar features to the actual Coloc network. These therefore included same or similar number of seeds (9 seeds) to the Coloc protein network and were matched to the mendelian protein network to quantify the number of mendelian proteins able to interact with the random seeds’ interactome. C, Nodes highlighted in yellow (Coloc proteins connected to mendelian Parkinson disease (PD) proteins and internodes) were used to run functional enrichment. The most specific terms of enrichment are reported in the table with their adjusted *P* value, gene ontology (GO) term identifier, and name. The proteins associated with the enrichment of the terms reported in tables are circled in red.

The protein products of the 11 candidate genes were interconnected (principally through a second-degree level of connection) with several proteins that are also relevant for mendelian forms of PD and parkinsonism (Figure 2A). The number of connections to known PD and parkinsonism genes (n = 9) was significantly higher than expected by chance (*P* < 1 × 10^−3^) based on a random simulation of 1000 control networks (Figure 2B). This result suggests a disease-specific and consistent interaction between protein products of our candidate genes and mendelian PD and parkinsonism genes.

Because proteins that interact together are likely to share similar functions, we investigated whether there were shared pathways and biological processes associated with the core composed of our candidate genes and mendelian genes (input list for enrichment in eTable 7 in Supplement 8). These results suggest that there is an enrichment of proteins involved in or regulating the ERBB receptor tyrosine protein kinase signaling pathways (Figure 2C; eTable 8 in Supplement 9).

## Discussion

With the increasing number of GWASs, our ability to map disease-associated variants exceeds our ability to interpret their biological function. Here, we have performed a comprehensive analysis by colocalization of eQTL and GWAS signals in PD, using a recent PD GWAS. We have integrated these data with publicly available brain eQTL data sets (Braineac and GTEx). This multilayered approach has identified 11 genes that we postulate underlie PD risk. Of these, 5 are associated with gene expression regulation, and 6 are associated with alternative splicing. We found evidence of methylation regulation for 3 of these candidate genes.

Of the candidate genes presented here, *CD38* is associated with insulin regulation, emphasizing a possible role of glucose metabolism in PD.^29^ This finding is supported by recent work indicating an association between body mass index and PD, and a randomized clinical trial of exenatide, a glucagon-like peptide 1 receptor agonist, as a disease-modifying agent for PD.^30,31^ Furthermore, a role for *CD38* in regulating neuroinflammation, especially in glial cells, has been proposed, which is consistent with the enrichment of *CD38* in astrocytes in the cell-type specificity analysis.^32^ The data from this study also reinforce *RAB29* as a key candidate for the chromosome 1q32 locus association. Recent studies providing further functional evidence linking *RAB29* (also known as *RAB7L1*) to *LRRK2*, and implicating *RAB29* as a substrate for *LRRK2* kinase activity, also support this designation.^33,34,35^ Furthermore, the candidate genes *CTSB* and *GALC*, whose dysfunction is linked to lysosomal storage disorders such as Krabbe disease, are consistent with the findings on the role of lysosomal pathways in PD.^36,37,38^ In the *GPNMB/NUPL2* locus, the PD GWAS results suggest only 1 independent signal, while the results presented in this article nominate both *GPNMB* (gene level) and *NUPL2* (splicing) with strong PPI evidence connecting both to mendelian or sporadic risk genes. This could be explained by the true causal gene being 1 of the 2, a single mechanism mediated through the associations with both genes, or potentially yet undetected independent PD GWAS signals at the locus associated with independent risk genes.

The WGCNA and WPPINA approaches allow prioritization of genes as a global functional unit. The WGCNA analysis suggested that 3 of the Coloc-prioritized genes (*NUPL2*, *TMEM163*, and *ZRANB3*) may be relevant for supporting functions associated with the ubiquitin proteasome system, neuronal development, and the chemical transmission at the synapse. The WPPINA analysis indicated that the proteins encoded by the Coloc-prioritized genes interact with mendelian PD and parkinsonism proteins, suggesting the existence of a common functional unit of genes and proteins—associated with the ERBB signaling pathways—that increases the risk for developing sporadic as well as familial PD.

### Limitations and Strengths

This study has some limitations. It considered only *cis*-QTLs, owing to the current challenges in robustly quantifying *trans*-QTLs. Furthermore, this study considered only QTLs in the brain and no other tissues. The Coloc analysis applied here assumes that the true causal variant underlying the disease has been captured in both the GWAS and eQTL data sets. The PD GWAS data used here have been imputed to the latest Haplotype Reference Consortium panel (version 1.1), and the genotypes in the GTEx data are generated with whole-genome sequencing, maximizing the chances of this assumption being met. However, the Braineac data used here have been imputed to 1000 Genomes phase 1, potentially reducing our power to replicate candidate genes in this data set. Another limitation in the colocalization tools used here is that they assume 1 independent signal for each gene at each locus for both the GWAS and QTL results. Finally, the methods used here cannot exclude pleiotropy, whereby a disease-causing SNV is associated with the regulation of an unrelated gene via a separate pathway.

Although the high degree of overlap between the GTEx and Braineac-derived results is encouraging, there were some inconsistencies in the implicated brain regions across the 2 data sets. This may be due to potential methodological differences in tissue collection, RNA extraction, platforms, and analysis pipelines. In addition, these differences might reflect divergent cell-type specificity of the expression and splicing effects.

This study also has some strengths. A key strength is that this is a large and comprehensive exploration of PD GWAS and eQTL data sets from the human brain. We replicated our Coloc results across 2 platforms, Braineac and GTEx, generated through microarray and RNA-seq, respectively, and performed additional validation using TWAS in the CommonMind Consortium dorsolateral prefrontal cortex data set. This has resulted in prioritizing 11 candidate causal genes for PD based on GWAS results to be further investigated biologically in different animal or cell models for PD. Furthermore, we are highlighting biological reasons for their likely functional association with PD pathogenesis. We acknowledge that further functional work will be required to mechanistically link these genes to PD, but the genetic and analytical approaches applied here suggest that these are the putative gene and genomic events underlying these risk loci.

## Conclusions

This study pairs GWAS with QTL data to discover candidate genes whose change in expression, splicing, or methylation are associated with the risk of PD. Furthermore, interaction network analyses highlight the functional pathways and cell types in which these candidate genes have an important role.

## Notes

Supplement 1. **eAppendix.** Supplementary Methods **eFigure 1.** Flowchart of Gene Expression Analysis (A) and Flowchart of Splicing Analysis (B) **eFigure 2.** Regional Association Plots of *GPNMB* With Braineac Data (A) and *RAB29* With GTEx Data (B) **eReferences.**

Supplement 2. **eTable 1.**

Supplement 3. **eTable 2.**

Supplement 4. **eTable 3.**

Supplement 5. **eTable 4.**

Supplement 6. **eTable 5.**

Supplement 7. **eTable 6.**

Supplement 8. **eTable 7.**

Supplement 9. **eTable 8.**

Supplement 10. **Nonauthor Collaborators. International Parkinson’s Disease Genomics Consortium (IPDGC) and United Kingdom Brain Expression Consortium (UKBEC) members**
