# Supplementary material for: Identification of Candidate Parkinson Disease Genes by Integrating Genome-Wide Association Study, Expression, and Epigenetic Data Sets
Source: JAMA Neurol. 2021 Feb 1;78(4):1–10. doi: 10.1001/jamaneurol.2020.5257 (PMC7851759; doi:10.1001/jamaneurol.2020.5257)
Supplement: Supplement 1. — eAppendix. Supplementary Methods eFigure 1. Flowchart of Gene Expression Analysis (A) and Flowchart of Splicing Analysis (B) eFigure 2. Regional Association Plots of GPNMB With Braineac Data (A) and RAB29 With GTEx Data (B) eReferences. [file jamaneurol-e205257-s001.pdf]

# Supplementary Online Content

Kia DA, Zhang D, Guelfi S, et al; United Kingdom Brain Expression Consortium (UKBEC); International Parkinson's Disease Genomics Consortium (IPDGC). Identification of candidate Parkinson disease genes by integrating genome-wide association study, expression, and epigenetic data sets. *JAMA Neurol.* Published online February 1, 2021. doi:10.1001/jamaneurol.2020.5257

**eAppendix.** Supplementary Methods
**eFigure 1.** Flowchart of Gene Expression Analysis (A) and Flowchart of Splicing Analysis (B)
**eFigure 2.** Regional Association Plots of *GPNMB* With Braineac Data (A) and *RAB29* With GTEx Data (B)
**eReferences.**

This supplementary material has been provided by the authors to give readers additional information about their work.

© 2021 Kia DA et al. *JAMA Network Open.*

**eAppendix.** Supplementary Methods

**DNA methylation quantification & quality control**

Post-mortem brain tissue from individuals with PD was obtained from both substantia nigra (n=129) and frontal cortex (n=134) individuals from the Parkinson's Disease UK Brain Bank. Genome wide methylation profiles of each DNA sample were by first bisulfite converting 1ug of DNA using the EZ DNA Methylation Kit (Zymo Research). DNAm was assayed using the Illumina Infinium HumanMethylation450 BeadChip (HM450), which targets over 485,000 CpGs genome-wide and 99% of reported RefSeq genes[1]. Samples were randomized across runs to minimize batch effects. BeadChips were scanned using the Illumina iScan System and raw intensity data was imported into Illumina GenomeStudio using the methylation module.

All subsequent DNAm data quality control procedures were performed in R using the recommended protocols for wateRmelon[2]. CpG sites were removed if they had a detection P-value > 5% in more than 1% of individuals and CpGs with a beadcount of less than three in more than 5% of individuals. Identity of samples was confirmed by matching DNAm gender with reported gender, and comparing SNP calls on the HM450 with genotyping calls. Non-autosomal probes, probes containing SNPs within the probe and cross-hybridising probes were subsequently removed[3]. Normalisation of DNAm data was performed using the "dasen" function, which performs background adjustment of methylated and unmethylated intensities and subsequent quantile normalisation of methylated and unmethylated Type 1 and Type II intensities independently.

**Genotyping and quality control for mQTL analysis**

DNA extracted from each sample was genotyped using the Illumina Human Exome Core array with additional custom content of approximately 27,000 SNPs which resulted in the genotyping of a total of >500,000 SNPs. SNPs were removed using the following filters using PLINK1.9; MAF<5%, genotyping rate <5%, HWE < 1E-05. Individuals were removed if overall genotyping rates were < 95% or were genetically related with PIHAT>0.125[4]. Eigenstrat was used to assess population substructure after which individuals were removed if they were more than 2 standard deviations away from the mean of the first 10 principle components[5]. Prior to imputation the genotypes were aligned to the Haplotype Reference Consortium (HRC) genotype reference panel using a publicly available perl script (http://www.well.ox.ac.uk/~wrayner/tools/). After alignment, genotypes were recoded in vcf format using PLINK1.9 and sorted using VCFtools[6]. Genotypes were uploaded to the Michigan Imputation Server (https://imputationserver.sph.umich.edu/index.html). Pre-imputation haplotype phasing was performed using Eagle and imputation was performed using Minimac3 (Sayantan Das et al, 2016) using the HRCv1.1 reference panel[7-9]. SNPs were retained with an imputation quality score >0.8 and maf>5%.

**mQTL Analysis**

© 2021 Kia DA et al. *JAMA Network Open.*

After DNAm and genotype quality control, data was available for 134 individuals. Cis PDmQTLs were defined as correlations between the target PD SNP genotype and DNAm levels of CpGs within a 500kb window of the SNP base position. mQTL analyses were performed using R package MatrixEQTL[10]. Linear models were fitted to test whether DNAm beta-values for each CpG were predicted by SNP genotypes. We included covariates for age at death, gender, population stratification, batch and post-mortem interval. We retained the strongest SNP-CpG pair at a 5% false discovery rate to be used in downstream analyses. CpGs were subsequently mapped to genes if they were within 10kb of the gene transcription start / end base position according to HG19 coordinates.

**Calculation of DNA methylation Fusion weights for TWAS**

FUSION weights for DNA methylation in the dorsolateral prefrontal cortex and substantia nigra were generated in-house using methylation and genotype data in up to 134 individuals using standard parameters of FUSION.compute_weights.R (http://gusevlab.org/projects/fusion/). After excluding CpGs that were not heritable ($p>0.01$) or failed to converge, FUSION-CpG weights were available for 37,460 and 36,620 probes in the frontal cortex and substantia nigra respectively. In order to biologically interpret the DNA methylation results, a CpG was assigned a protein-coding gene if it was within 10kb of the gene transcription start/end base position according to HG19 coordinates.

**Literature-derived PPI networks**

We extracted currently known protein interactors (PPIs) for the proteins (seeds) encoded by the genes prioritized in this manuscript (coloc protein network). PPIs were identified for each seed protein based upon entries in the following databases within the IMEX consortium[11]: APID Interactomes, BioGrid, bhf-ucl, InnateDB, InnateDB-All, IntAct, mentha, MINT, InnateDB-IMEx, UniProt, and MBInfo by means of the "PSICQUIC" R package (version 1.15.0 by Paul Shannon, http://code.google.com/p/psicquic/). For 2 of the seeds (*TMEM163* and *NEK*) no human protein interaction data was available thus they were excluded form the analysis. After downloading PPIs (21 January 2018), Protein IDs were converted to uniprot and Entrez IDs. All databases were merged after removal of TrEMBL, non-protein interactors (e.g. chemicals), obsolete Entrez and Entrez matching to multiple Swiss-Prot identifiers. All PPIs underwent quality control (QC) following the weighted protein-protein interaction network analysis (WPPINA) pipeline as previously described to remove: i) all non-human annotations, and; ii) all poor quality annotations[12]. The interactions were then scored taking into consideration the number of different publications reporting the interaction, and the number of different methods reporting the interaction. All the interactors with a final score $\leq 2$ were discarded to control for replication and reduce false positive rate. Of note, UBC (coding for the protein ubiquitin) was removed due to the pervasive nature of covalent ubiquitylation of proteins as part of the ubiquitin/proteasome degradation pathway. A similar network (Mendelian protein network) was prepared for Mendelian, Parkinson's/parkinsonism proteins (*SNCA, LRRK2, GBA, SMPD1, VPS35, DNAJC13, PINK1, PRKN, DJ1, FBXO7, SYNJ1, DNAJC6, PRKRA, C19Orf12, PANK2, SPG11, RAB39B, ATP13A2, PLA2G6* and *WDR45*) and for 118 genes with a negative score in the coloc analysis (the latter being used as a negative

© 2021 Kia DA et al. *JAMA Network Open.*

control protein network, Supplementary Table 1. The final networks were visualized through the freely available Cytoscape 3.5.0 software[13]. Functional enrichment was on the relevant genes prioritized through WPPINA was performed using g:Profiler (doi: 10.1093/nar/gkw199) on the 8th February 2018 allowing for Gene Ontology (GO) Biological Processes (BP) terms to be queried.

© 2021 Kia DA et al. *JAMA Network Open.*

**eFigure 1.** Flowchart of Gene Expression Analysis (A) and Flowchart of Splicing Analysis (B)

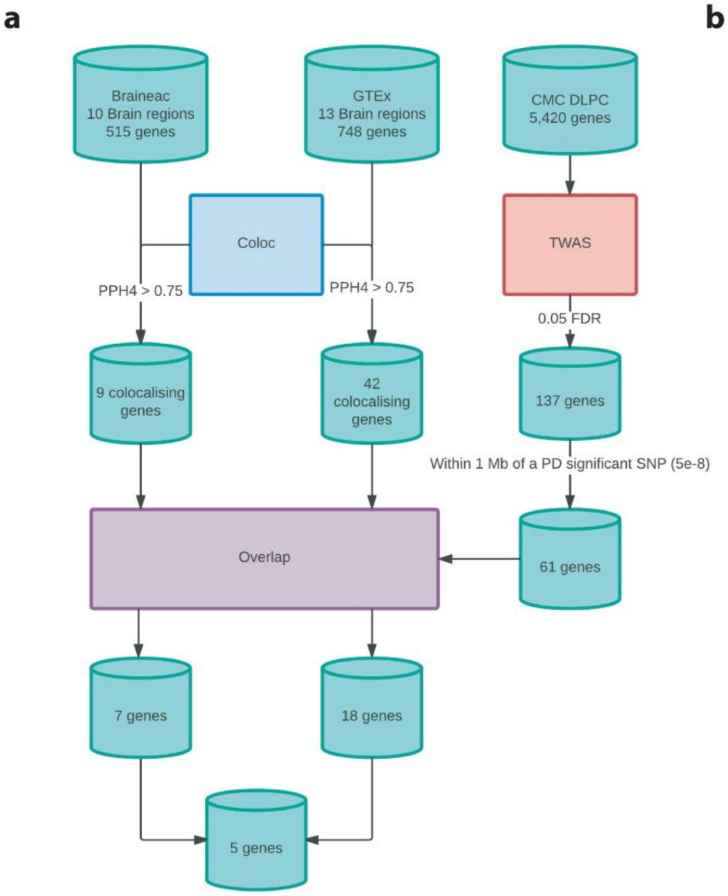


**eFigure 1. A:** Flowchart of gene expression analysis. Overall, 5 genes replicated across GTEx and Braineac, and in the TWAS analysis. CMC = CommonMind Consortium; DLPC = dorsolateral prefrontal cortex; TWAS = transcriptome-wide association study. **B:** Flowchart of splicing analysis. Overall, 6 genes replicated across Coloc splicing analysis in Braineac and TWAS sQTL analysis. For Coloc, the splicing analysis consisted of identifying genes with evidence for colocalization at the level of at least one exon (exon PPH4 > 0.75) and evidence against colocalization for the expression of the whole gene (gene PPH3 > PPH4). CMC = CommonMind Consortium; DLPC = dorsolateral prefrontal cortex; TWAS = transcriptome-wide association study.

**eFigure 2.** Regional Association Plots of *GPNMB* With Braineac Data (A) and *RAB29* With GTEx Data (B)

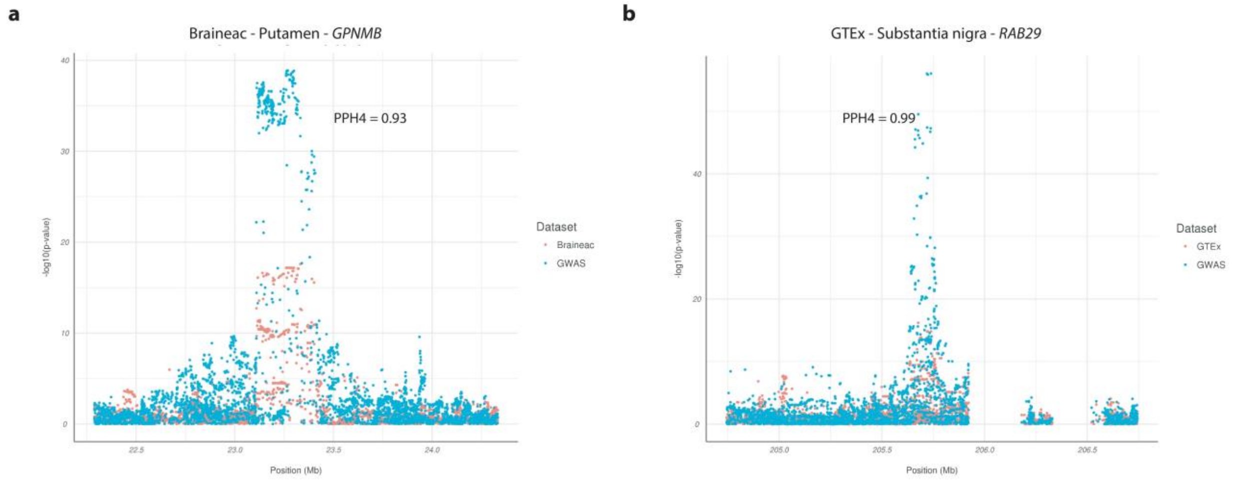


e**Figure 2:** Regional association plots of *GPNMB* with Braineac data **A:** and *RAB29* with GTEx data **B:** The -log10 p-values are presented on the association of each SNP with gene expression (orange) and risk of PD (green), illustrating the likely colocalization of the signals and the likely presence of a shared causal variant.

**eReferences**


1. Price EM, Cotton AM, Lam LL, et al. Additional annotation enhances potential for biologically-relevant analysis of the Illumina Infinium HumanMethylation 450 BeadChip array. *Epigenetics Chromatin.* 2013;6(1):4.
2. Pidsley R, CC YW, Volta M, Lunnon K, Mill J, Schalkwyk LC. A data-driven approach to preprocessing Illumina 450K methylation array data. *BMC genomics.* 2013;14:293.
3. Chen YA, Lemire M, Choufani S, et al. Discovery of cross-reactive probes and polymorphic CpGs in the Illumina Infinium HumanMethylation450 microarray. *Epigenetics.* 2013;8(2):203-209.
4. Chang CC, Chow CC, Tellier LC, Vattikuti S, Purcell SM, Lee JJ. Second-generation PLINK: rising to the challenge of larger and richer datasets. *Gigascience*. 2015;4(1):s13742-13015-10047-13748.
5. Price AL, Patterson NJ, Plenge RM, Weinblatt ME, Shadick NA, Reich D. Principal components analysis corrects for stratification in genome-wide association studies. *Nat Genet*. 2006;38(8):904-909.
6. Danecek P, Auton A, Abecasis G, et al. The variant call format and VCFtools. *Bioinformatics*. 2011;27(15):2156-2158.
7. Das S, Forer L, Schönherr S, et al. Next-generation genotype imputation service and methods. *Nat Genet*. 2016;48(10):1284-1287.
8. Loh P-R, Danecek P, Palamara PF, et al. Reference-based phasing using the Haplotype Reference Consortium panel. *Nat Genet*. 2016;48(11):1443.
9. McCarthy S, Das S, Kretzschmar W, et al. A reference panel of 64,976 haplotypes for genotype imputation. *Nat Genet*. 2016;48(10):1279-1283.
10. Shabalin AA. Matrix eQTL: ultra fast eQTL analysis via large matrix operations. *Bioinformatics.* 2012;28(10):1353-1358.
11. Orchard S, Kerrien S, Abbani S, et al. Protein interaction data curation: the International Molecular Exchange (IMEx) consortium. *Nat Methods*. 2012;9(4):345-350.
12. Ferrari R, Lovering RC, Hardy J, Lewis PA, Manzoni C. Weighted protein interaction network analysis of frontotemporal dementia. *J Proteome Res.* 2017;16(2):999-1013.
13. Shannon P, Markiel A, Ozier O, et al. Cytoscape: a software environment for integrated models of biomolecular interaction networks. *Genome Res.* 2003;13(11):2498-2504.


© 2021 Kia DA et al. *JAMA Network Open.*
