# Supplementary material for: Identification of Candidate Parkinson Disease Genes by Integrating Genome-Wide Association Study, Expression, and Epigenetic Data Sets
Source: JAMA Neurol. 2021 Feb 1;78(4):1–10. doi: 10.1001/jamaneurol.2020.5257 (PMC7851759; doi:10.1001/jamaneurol.2020.5257)
Supplement: Supplement 2. — eTable 1. [file jamaneurol-e205257-s002.pdf]

Kia DA, Zhang D, Guelfi S, et al; United Kingdom Brain Expression Consortium (UKBEC); International Parkinson's Disease Genomics Consortium (IPDGC). Identification of candidate Parkinson disease genes by integrating genome-wide association study, expression, and epigenetic data sets. JAMA Neurol. Published online February 1, 2021. doi:10.1001/jamaneurol.2020.5257

gene_ID

GLB1L3

PPHLN1 HSPC206 HSPC232

PARP1 ADPRT PPOL

WDR11 BRWD2 KIAA1351 WDR15

RNF123 KPC1 FP1477

POU5F1 OCT3 OCT4 OTF3

H3F3A H3.3A H3F3 PP781 H3F3B H3.3B

TCTN2 C12orf38 TECT2

QPRT

DNMT3B

TACC3 ERIC1

TIMM13 TIM13B TIMM13A TIMM13B

GJC1 GJA7

INO80E CCDC95

LRRK2 PARK8

NOS1AP CAPON KIAA0464

MLK4 KIAA1804

KTN1 CG1 KIAA0004

NCLN

RNF5 G16 NG2 RMA1

ERCC8 CKN1 CSA

RXRB NR2B2

ITPA C20orf37 My049 OK/SW-cl.9

WNT3 INT4

BRD2 KIAA9001 RING3

RBBP5 RBQ3

SKIV2L DDX13 SKI2W SKIV2 W

KANSL1 CENP-36 KIAA1267 MSL1V1 NSL1

MAPK3 ERK1 PRKM3

G3BP2 KIAA0660

COL11A2

NUP54

BAK1 BAK BCL2L7 CDN1

ZGRF1 C4orf21

FLOT1

NMT1 NMT

TGFB1I1 ARA55

NTPCR C1orf57

RPS18 D6S218E

SEPT11

ACBD4 HMFT0700

NBR1 1A13B KIAA0049 M17S2 MIG19

SNAP47 C1orf142 HEL170 SVAP1

MICA PERB11.1

RBL2 RB2

UVSSA KIAA1530

SMG5 EST1B KIAA1089

HLA-DPA1 HLA-DP1A HLASB

NAAA ASAHL PLT

RND2 ARHN RHO7

DSTYK KIAA0472 RIP5 RIPK5 SGK496 HDCMD38P

SETD8 KMT5A PRSET7 SET07 SET8

BMP4 BMP2B DVR4

THBS3 TSP3

GNA15 GNA16

RNF149 DNAPTP2

AKTIP FTS

RNF24

PHYHIP DYRK1AP3 KIAA0273

IL1RL1 DER4 ST2 T1

APC2 APCL

TNFRSF10C DCR1 LIT TRAILR3 TRID UNQ321/PRO366

C4A CO4 CPAMD2

HLA-DQA1

VAT1

REEP6 C19orf32 DP1L1

ABCC5 MRP5

NUDT18 MTH3

MAEA EMP HLC10 PIG5

CHMP7

LEMD2

KNOP1 C16orf88 FAM191A TSG118

DCAKD

MTUS1 ATBP ATIP GK1 KIAA1288 MTSG1

DNM3 KIAA0820

FAM221A C7orf46

CCNG2

PPP3CC CALNA3 CNA3

EIF2A CDA02 MSTP004 MSTP089

GPX1

PRICKLE1 RILP

RHOF ARHF RIF

PEBP4 CORK1 UNQ1933/PRO4408

NANOS1 NOS1

CNTN1

HLA-DQB2 HLA-DXB

ART3 TMART

ATG14 ATG14L KIAA0831

BTN3A2 BT3.2 BTF3 BTF4

KLHL24 DRE1

CRIPAK

THOP1

KLHDC8A

HLA-DPB1 HLA-DP1B

NPM2

GTF2H4

TCF7 TCF1

DBF4B ASKL1 DRF1

MSTO1 LST005 SLTP005

HLA-C D6S204 HLA-JY3 HLAC

BTN2A2 BT2.2 BTF2

IL18R1 IL1RRP

SPATA7 HSD3

PIWIL2 HILI

HLA-DMA DMA RING6

HLA-DRB5

HLA-DQB1 HLA-DQB

HLA-DOA HLA-DNA HLA-DZA

APOA1BP AIBP YJEFN1

C5orf64

PIGG GPI7 UNQ1930/PRO4405

ZNF554

TMEM129

WDR5B

SFXN4 BCRM1

ZNF843

ARL4D ARF4L

TMA7 CCDC72 HSPC016 HSPC330
