# Supplementary material for: Identification of Candidate Parkinson Disease Genes by Integrating Genome-Wide Association Study, Expression, and Epigenetic Data Sets
Source: JAMA Neurol. 2021 Feb 1;78(4):1–10. doi: 10.1001/jamaneurol.2020.5257 (PMC7851759; doi:10.1001/jamaneurol.2020.5257)
Supplement: Supplement 10. — Nonauthor Collaborators. International Parkinson’s Disease Genomics Consortium (IPDGC) and United Kingdom Brain Expression Consortium (UKBEC) members [file jamaneurol-e205257-s0010.pdf]

Group Name: The United Kingdom Brain Expression Consortium (UKBEC) and the International Parkinson’s Disease Genomics Consortium (IPDGC) members							
First Name and Middle Initial(s)	Last Name	Suffix	Academic Degrees	Institution	Location (city, state/province, country)	Role or Contribution, eg, chair, principal investigator	Subgroup, eg, Steering Committee
Alastair J.	Noyce						
Rauan	Kaiyrzhanov						
Ben	Middlehurst						
Demis A.	Kia						
Manuela	Tan						
Henry	Houlden						
Huw R.	Morris						
Helene	Plun-Favreau						
Peter	Holmans						
John	Hardy						
Daniah	Trabzuni						
Jose	Bras						
John Quinn	PhD						
Kin Y.	Mok						
Kerri J.	Kinghorn						
Kimberley	Billingsley						
Nicholas W.	Wood						
Patrick	Lewis						
Sebastian	Schreglmann						
Rita	Guerreiro						
Ruth	Lovering						
Lea	R'Bibo						
Claudia	Manzoni						
Mie	Rizig						
Mina	Ryten						
Sebastian	Guelfi						
Valentina	Escott-Price						
Viorica	Chelban						
Thomas	Foltynie						
Nigel	Williams						
Alexis	Brice						

First Name and Middle Initial(s)	Last Name	Suffix	Academic Degrees	Institution	Location (city, state/province, country)	Role or Contribution, eg, chair, principal investigator	Subgroup, eg, Steering Committee
Fabrice	Danjou						
Suzanne	Lesage						
Jean-Christophe	Corvol						
Maria	Martinez						
Claudia	Schulte						
Kathrin	Brockmann						
Javier	Simón-Sánchez						
Peter	Heutink						
Patrizia	Rizzu						
Manu	Sharma						
Thomas	Gasser						
Aude	Nicolas						
Mark R.	Cookson						
Sara	Bandres-Ciga						
Cornelis	Blauwendraat						
David W.	Craig						
Faraz	Faghri						
J. Raphael	Gibbs						
Dena G.	Hernandez						
Kendall	Van Keuren-Jensen						
Joshua M.	Shulman						
Hampton L.	Leonard						
Mike A.	Nalls						
Laurie	Robak						
Steven	Lubbe						
Steven	Finkbeiner						
Niccolo E.	Mencacci						
Codrin	Lungu						
Andrew B	Singleton						
Sonja W.	Scholz						
Xylena	Reed						
Roy N.	Alcalay						

First Name and Middle Initial(s)	Last Name	Suffix	Academic Degrees	Institution	Location (city, state/province, country)	Role or Contribution, eg, chair, principal investigator	Subgroup, eg, Steering Committee
Ziv	Gan-Or						
Guy A.	Rouleau						
Lynne	Krohn						
Jacobus J.	van Hilten						
Johan	Marinus						
Astrid D.	Adarmes-Gómez						
Miquel	Aguilar						
Ignacio	Alvarez						
Victoria	Alvarez						
Francisco	Javier Barrero						
Jesús A.	Bergareche Yarza						
Inmaculada	Bernal-Bernal						
Marta	Blazquez						
Marta	Bonilla-Toribio						
Juan A.	Botía						
María T.	Boungiorno						
Dolores	Buiza-Rueda						
Ana	Càmara						
Fátima	Carrillo						
Mario	Carrión-Claro						
Debora	Cerdan						
Jordi	Clarimón						
Yaroslau	Compta						
Monica	Diez-Fairen						
Oriol	Dols-Icardo						
Jacinto	Duarte						
Raquel	Duran						
Francisco	Escamilla-Sevilla						
Mario	Ezquerra						
Cici	Feliz						
Manel	Fernàndez						
Rubén	Fernàndez-Santiago						

First Name and Middle Initial(s)	Last Name	Suffix	Academic Degrees	Institution	Location (city, state/province, country)	Role or Contribution, eg, chair, principal investigator	Subgroup, eg, Steering Committee
Ciara	Garcia						
Pedro	García-Ruiz						
Pilar	Gómez-Garre						
Maria J.	Gomez Heredia						
Isabel	Gonzalez-Aramburu						
Ana G.	Pagola						
Janet	Hoenicka						
Jon	Infante						
Adriano	Jimenez-Escrig						
Jaime	Kulisevsky						
Miguel A.	Labrador-Espinosa						
Jose Luis	Lopez-Sendon						
Adolfo López de Mu	Arregui						
Daniel	Macias						
Irene Martínez	Torres						
Juan	Marín						
Maria Jose	Marti						
Juan Carlos	Martínez-Castrillo						
Carlota	Mèndez-del-Barrio						
Manuel Menéndez	González						
Marina Mata	Adolfo Mínguez						
Pablo	Mir						
Elisabet Mondragor	Rezola						
Esteban	Muñoz						
Javier	Pagonabarraga						
Pau	Pastor						
Francisco Perez	Errazquin						
Teresa	Perińán-Tocino						
Javier	Ruiz-Martínez						
Clara	Ruz						
Antonio Sanchez	Rodriguez						
María	Sierra						

First Name and Middle Initial(s)	Last Name	Suffix	Academic Degrees	Institution	Location (city, state/province, country)	Role or Contribution, eg, chair, principal investigator	Subgroup, eg, Steering Committee
Esther	Suarez-Sanmartin						
Cesar	Tabernero						
Juan Pablo	Tartari						
Cristina	Tejera-Parrado						
Eduard	Tolosa						
Francesc	Valldeoriola						
Laura	Vargas-González						
Lydia	Vela						
Francisco	Vives						
Alexander	Zimprich						
Lasse	Pihlstrom						
Mathias	Toft						
Sulev	Koks						
Pille	Taba						
Sharon	Hassin-Baer						
John	Hardy						
Michael	Weale						
Mina	Ryten						
Daniah	Trabzuni						
Adaikalavan	Ramasamy						
Colin	Smith						
Manuel Sebastian	Guelfi						
Karishma	D'sa						
Paola	Forabosco						
Juan A.	Botiá						
